# Optimized Multiplex Detection of 7 KRAS Mutations by Taqman Allele-Specific qPCR

**DOI:** 10.1371/journal.pone.0163070

**Published:** 2016-09-15

**Authors:** Andrea Orue, Manuel Rieber

**Affiliations:** IVIC, Tumor Cell Biology Laboratory, Apartado 21827, Caracas, 1020A, Venezuela; Rutgers, the State Univesity of New Jersey, UNITED STATES

## Abstract

Establishing the *KRAS* mutational status of tumor samples is essential to manage patients with colorectal or lung cancer, since these mutations preclude treatment with monoclonal anti-epidermal growth factor receptor (EGFR) antibodies. We report an inexpensive, rapid multiplex allele-specific qPCR method detecting the 7 most clinically relevant KRAS somatic mutations with concomitant amplification of non-mutated KRAS in tumor cells and tissues from CRC patients. Positive samples evidenced in the multiplex assay were further subjected to individual allele-specific analysis, to define the specific mutation. Reference human cancer DNA harbouring either G12A, G12C, G12D, G12R, G12S, G12V and G13D confirmed assay specificity with ≤1% sensitivity of mutant alleles. KRAS multiplex mutation analysis usefulness was also demonstrated with formalin-fixed paraffin embedded (FFPE) from CRC biopsies. Conclusion. Co-amplification of non-mutated DNA avoided false negatives from degraded samples. Moreover, this cost effective assay is compatible with mutation detection by DNA sequencing in FFPE tissues, but with a greater sensitivity when mutant DNA concentrations are limiting.

## Introduction

Colorectal cancer (CRC) is the third most common cancer diagnosed in men and the second most common in women worldwide [1]. Cetuximab and Panitumumab are monoclonal antibodies directed against the epidermal growth factor receptor (EGFR) clinically used for the molecular targeted therapy on colorectal carcinoma [2]. Oncogenic KRAS mutations are well established negative predictors of response to anti-EGFR therapies, because they generate a constitutively active protein, leading to stimulus independent, persistent activation of downstream effectors, such as the RAF/mitogen-activated protein kinase kinase (MEK)/extracellular signal-regulated kinase (ERK) cascade, [3,4,5]. Phosphoprotein activation of several MAPK signaling components frequently is stronger in KRAS-mutants than in any other tumor groups. The mutant KRAS associated oncogenic activation is observed in 35% to 40% of colorectal carcinomas, and most cases have mutations in codons 12 (80%) and 13 (15%) of exon 2 [6,7,8]. Mutations in other positions, such as codons 61, 117, 146 and 154, are much less frequent, representing only ~1% of all KRAS gene mutations [9,4]. The European Medicines Agency (EMA), the National Comprehensive Cancer Network (NCCN), the American Society of Clinical Oncology (ASCO) and the US Food and Drug Administration (FDA) recommend that treatment of cetuximab and panitumumab to target EGFR, requires that CRC patients posses a wild type KRAS. Hence, simple and sensitive screening for KRAS mutations prior to treatment with an anti-EGFR antibody therapy is indispensable [2,10]. Early detection in clinically available tissue is difficult in cases with low abundance mutations relative to wild type DNA. This requires laborious, expensive, time consuming techniques of microdissection to isolate the tumor cells prior performing molecular analysis, unsuitable for routine pathological analysis [11]. As such, the detection and identification of minority alleles present at low abundance is a challenge and dependent upon the accuracy and sensitivity of the molecular techniques and by the methods employed, limiting the diagnostic potential of such rare mutations.

Many molecular techniques have been developed for detecting KRAS mutations, each with its advantages and disadvantages, differ regarding cost, test duration, sensitivity, specifity, reproducibility according to the issue tested (frozen or formalin fixed, paraffin embedded tissue), capacity to quantify the mutated alleles, and ability to detect new mutations [12,13,14].

Among these methods, Sanger sequencing, is considered a gold standard, but has low sensitivity, requiring approximately 10–30% mutated KRAS alleles in a wild type background [15,12]. Since PCR will amplify all alleles with approximately equal efficiency comparable to their initial concentrations [16], it is desirable to selectively decrease the wild type amplification [2,17]. Allele-specific PCR, also known as amplification refractory mutation system (ARMS), is based on the principle that amplification is efficient when the 3′ terminal base of the primer matches the template, whereas amplification is inefficient or even nonexistent when there is a mismatch [18,19]. Combining allele-specific PCR and real-time PCR techniques based on TaqMan probes allows high-throughput and sensitive detection of mutations with an improved interpretation of PCR results. Assays based on this strategy have been developed for clinical applications [20–25] and several commercial kits have been developed for clinical applications, such as Therascreen KRAS RGQ PCR kit (Qiagen) and Cobas KRAS kit (TaqMelt PCR) [21,22,25–30].

In this study, we developed a rapid, cost effective, multiplex allele specific assay high throughput for screening of the most common KRAS mutations in codons 12 and 13 in FFPE biopsies from cancer patients with high sensitivity. Our assay is based in a single tube multiplex qPCR using 7 allele specific primers that anneal specifically to a mutated DNA template. Positive samples evidenced in the multiplex assay were further subjected to individual allele-specific analysis, to confirm the specific mutation.

## Materials and Methods

### Reference DNA and clinical samples

We used genomic DNA of reference standard harboring KRAS wild-type DNA and seven point mutations in KRAS codons 12 and 13 (G12D, G12A, G12R, G12C, G12S, G12V, and G13D). The KRAS DNAs reference standards were from Horizon, Cambridge CB25 9PL, UK.

Formalin-fixed paraffin-embedded (FFPE) colorectal adenocarcinomas from 32 patients with colorectal carcinoma were collected from the Centre for Sanger Sequencing and Analysis (CESAN) of the Venezuelan Institute for Scientific Research (IVIC), Venezuela. The study was approved by the Ethics Committee of the Institute and the data of tissue samples were analyzed anonymously.

### Genomic DNA extraction from formalin- fixed paraffin-embedded samples

DNA was isolated from cultured cells by DNAzol–chloroform deproteinization as recommended by the manufacturer (Invitrogen). The Genomic DNA (gDNA) from the FFPE sections was isolated using a QIAamp DNA FFPE Tissue Kit (QIAGEN) according to the manufacturer’s instructions. Briefly, tumors were manually micro-dissected from paraffin-embedded blocks, and 10 μm thick sections were collected in a 1.5 ml tube. Paraffin was removed from the tissue blocks with xylene, and samples were air-dried. DNA quantity was determined by fluorometric quantitation in a Qubit 2.0 fluorimeter (Q32866, Invitrogen). DNA samples were stored at -20°C until needed.

### Mutation Detection by qPCR assay

Mutations in codons 12 and 13 of the KRAS gene (accession N° NG_007524) were determined by qPCR using allele specific primers (AsP) designed for each of seven mutations and a non-mutated (NM) region used as a reference amplicon [22] (S1 Table). The 3´ terminal base of each allele specific primer was adapted according to its corresponding mutation. In addition, an artificial mismatch at the penultimate or antepenultimate base was included in the allele specific primers to improve specificity [22] PCR reactions mixtures were carried out in 48-well plates in Eco Illumina Real Time PCR system (Illumina). Our uniplex reaction conditions included 0.35 μM forward primer, 0.35 μM reverse primer and 0.5 μM probe and 3 μl of DNA template of varying concentration. Reactions were performed in 1X TaqMan Genotyping Mix (Cat N° 4371355, Applied Biosystems, Foster City,Ca USA) in a 10 μl total volume. In contrast to a previous Taqman uniplex protocol using initial DNA denaturation 95°C for 10 minutes, 50 cycles of 90°C for 15 sec and 60°C for 60 sec [22], our multiplex qPCR similarly used a prior 95°C for 10 min step, but required optimization such as 50 cycles of 95°C for 30 sec and 60°C for 30 sec. Each sample was evaluated in triplicate. At least one negative control with no DNA (NTC) was included in every run.

### Multiplex Allele-Specific Assay (Mult-AsP)

Multiplex allele specific assays were performed according to the same protocol but using all seven allele specific primers for the identification of KRAS mutation, the same reverse primer and the Taqman probe in a single tube. These multiplex reactions included 0.4 μM of each allele specific forward primer, 0.55 μM reverse primer and 0.5 μM probe and 35 ng/μl DNA template with PCR conditions described above. For each template, the Cq values were recorded for reference PCR, each allele specific PCR and multiplex allele specific, and the corresponding ΔCq values were calculated (differences in threshold cycles between the allele-specific assay and the reference assay, and differences in threshold cycles between the multiplex allele specific and the reference assay).

### KRAS genotyping in clinical samples

The KRAS genotyping in clinical FFPE tissues also included the non-mutated reference amplicon (NM) and multiplex allele specific primers in the qPCR assay. The protocol for evaluation of clinical samples involved a first step the amplification simultaneous of both non-mutated reference amplicon and multiplex allele specific assay. When the qPCR for the non-mutated reference DNA was negative, the sample was not analyzed further. However, if the multiplex amplification was negative and the non-mutated amplicon positive, the sample is considered negative. In case of multiplex-positive clinical samples, these were subjected to seven individual PCR reactions KRAS mutants with allele specific primer for the identification of mutation. All samples were evaluated by triplicate.

### Sensitivity of multiplex allele specific assay

To establish the range of lowest detection limit of multiplex allele specific assay, the mutated DNA KRAS was mixed with a wild type DNA KRAS. The proportion of mutant DNA was gradually reduced to obtain decreasing ratios of mutant to wild-type DNA at 5%, 2.5%, 1.25%, 0.62%, 0.32% and 0.15%. Reactions were performed in triplicate with negative control well with no DNA (NTC).

## Results

### KRAS genotyping multiplex allele specific assay design

**Fig 1:**
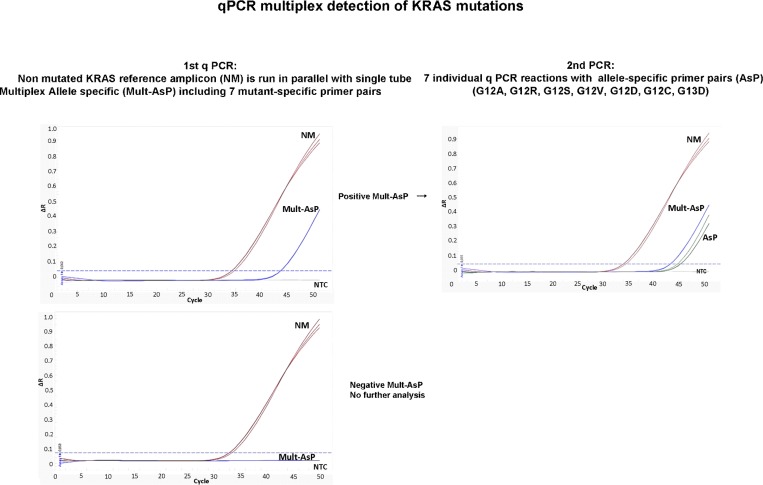
Design of multiplex qPCR for KRAS genotyping. In a first step was performed PCR to amplify the Non-Mutated reference amplicon KRAS (NM) and simultaneously Multiplex Allele specific (Mult-AsP). When both amplicons were amplified (NM and Mult-AsP), the sample was interpreted as positive and seven allele specific primer (AsP) PCR reactions for the specific identification of mutation were performed. If no amplicon is amplified in the Mult-AsP reaction but amplified in the non-mutated reference reaction, the sample was interpreted as negative or wild type.

**Fig 2:**
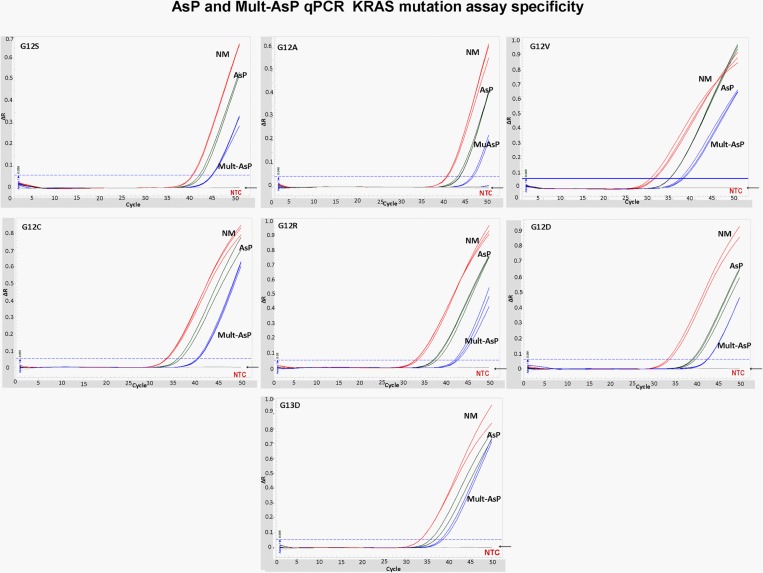
AsP and Mult-AsP PCR assays for analysis of the mutational status of *KRAS* codons 12 and 13 in reference samples. Genomic DNA of seven reference standards harboring KRAS mutations in codons 12 and 13, was used for AsP and Mult-AsP PCR assay. In all cases the qPCR assays contained the non-mutated reference control reaction (red line). Each reference DNA was amplified in AsP and Multi-AsP PCR (green and blue lines, respectively). In gray curves indicated NTC reaction.

**Fig 3:**
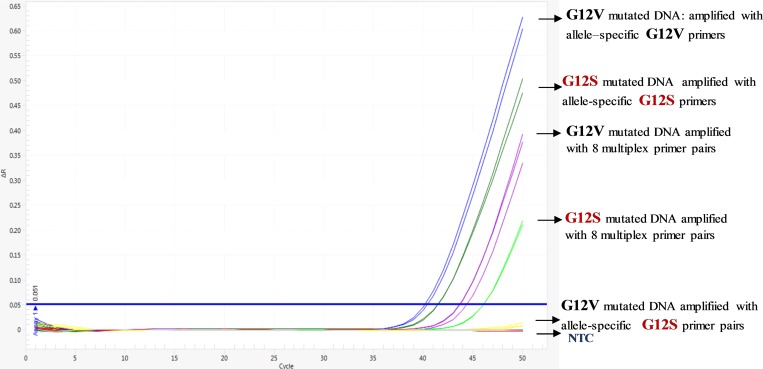
Allele-specific primer only amplify sequence-specific templates. Genomic DNA harbouring G12V or G12V KRAS mutations were used for AsP and Mult-AsP PCR assays, whenever indicated.

We developed a multiplex allele specific assay specific based in screen the 7 most common KRAS mutations in codons 12 and 13 for colorectal carcinoma, using an allele specific qPCR assay (see Fig 1). The first step involved a non- mutated reference amplicon (control reaction) concomitantly with multiplex allele specific assays. This was followed by uniplex confirmatory assays which gave reproducible results with no cross reactivity between allele specific primers combinations. Fig 2 shows amplification reactions for multiplex allele specific, DNA of each of the seven most frequent KRAS mutations with the respective. ΔCq values shown in Table 1. Furthermore, allele-specific primers only amplified sequence-specific templates, since uniplex qPCR using G12S specific primers did not amplify a G12V DNA, in contrast to the expected amplification of G12S DNA with its respective primers (Fig 3).

**Table 1:** ΔCq values of AsP and Mult-AsP qPCR assays.

	NM		AsP				Mult-AsP			
Mutation	$\bar{Cq}$	SD	$\bar{Cq}$	SD	$\bar{\Delta Cq}$	SD	$\bar{Cq}$	SD	$\bar{\Delta Cq}$	SD
**G12A**	40.27	0.09	43.05	0.29	2.78	0.29	46.23	0.23	5.96	0.16
**G12S**	39.61	0.13	41.38	0.35	1.77	0.35	44.00	0.07	4.39	0.10
**G12R**	33.01	0.31	37.37	0.45	4.37	0.45	42.32	0.38	9.32	0.34
**G12C**	33.36	0.09	36.29	0.42	2.93	0.42	40.34	0.15	6.98	0.12
**G12V**	30.37	0.52	34.26	0.04	3.89	0.04	37.11	0.39	6.74	0.45
**G12D**	33.38	0.32	39.38	0.38	6.10	0.38	42.61	0.05	9.23	0.18
**G13D**	33.64	0.15	36.47	0.82	2.83	0.82	39.04	0.98	5.40	0.74

$\bar{Cq}$ represents Cq mean and $\bar{\Delta Cq}$ represents ΔCq mean. No qPCR amplification was detected with the NTC (non-template controls)

### KRAS genetic mutation analysis using the FFPE samples of colorectal carcinoma

**Fig 4:**
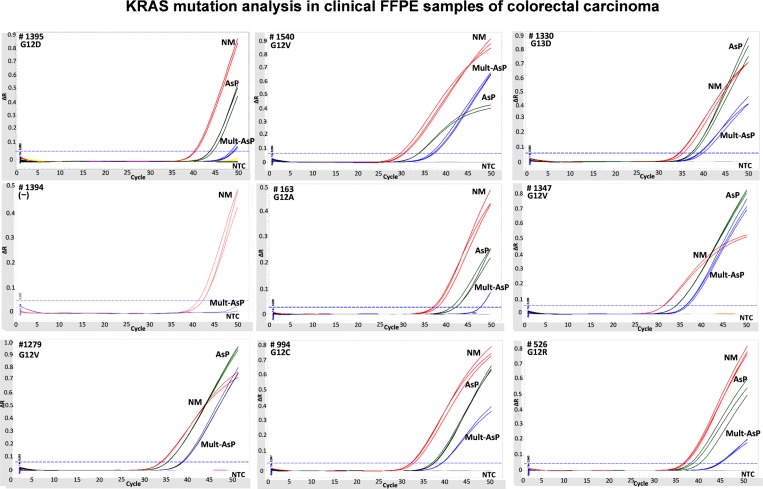
KRAS multiplex mutation analysis with colorectal carcinoma FFPE. Genomic DNA from FFPE tissues were used for AsP and Mult-AsP PCR assay. In all cases the qPCR assays contained the non-mutated reference control reaction (red line). Each DNA was amplified in AsP and Multi-AsP PCR (green and blue lines, respectively). In gray curves indicated NTC reaction.

The multiplex allele specific assay, performed on the 32 CRC FFPE samples, identifying mutations in 21 samples. The amplifications curves (representative’s samples) are shown in Fig 4. Reactions were run in triplicate and the mean +/- SD ΔCt values are shown in Table 2. The comparison of multiplex allele specific assays and Sanger sequencing is presented in Table 2. In this qPCR study, we identified that 22 of 30 cases analyzed for KRAS gave amplifiable PCR products by the multiplex allele specific approach. 6 cases gave no amplifiable products with Sanger sequencing but showed KRAS mutations by our multiplex assay confirmed by our uniplex qPCR. The discordant result between Sanger and allele-specific multiplex assays, is likely linked to the low tumor to non-neoplastic cell ratio and the higher analytical sensitivity of multiplex assay.No amplifiable PCR product was evident in 8 other cases either by Sanger sequencing or our multiplex/ allele specific protocol. 7 of these samples originated from provincial dispensaries, which were inadequately processed because of limited pathological proficiency, perhaps leading to excessive DNA degradation, as reported by others (17). This is believed because no amplification was seen from these samples using non mutated reference DNA primers. In all of these 8 cases the negative samples were re-tested up to 3 times both Sanger sequencing and reference non-mutated assay as reaction control. Other samples were considered degraded because of their inability to amplify the non-mutated reference amplicon.

**Table 2:** KRAS mutation analysis relative to NM reference DNA in colorectal carcinoma FFPE tissues.

				qPCR	AsP		Mult-AsP		Sanger sequencing
# FFPE	Age	Gender	Tumor Site	Mutation	ΔCq	SD	ΔCq	SD	Mutation
**1395**	60	F	Metastatic	**G12D**	2.99	0.41	7.94	0.35	**G12D**
**1330**	45	F	Metastatic	**G13D**	2.40	0.24	4.37	0.12	**G13D**
**1540**	67	F	Primary	**G12V**	3.57	0.07	6.74	0.09	**G12V**
**1777**	55	F	Metastatic	**G12C**	0.86	1.18	3.08	1.10	Undetermined
**1279**	65	M	Primary	**G12V**	1.49	0.04	4.84	0.03	**G12V**
**1347**	40	F	Metastatic	**G12V**	2.86	0.04	5.17	0.20	**G12V**
**163**	61	F	Primary	**G12A**	4.40	0.59	10.26	0.02	**G12A**
**994**	59	M	Metastatic	**G12C**	4.78	0.27	6.71	0.10	**G12C**
**526**	44	F	Primary	**G12R**	2.30	1.10	7.88	0.20	**G12R**
**299**	50	F	Primary	**G12R**	3.06	0.80	7.75	1.2	**G12R**
**1739**	55	F	Metastatic	**G12D**	2.46	0.35	5.80	0.45	Undetermined
**1778**	64	F	Primary	**G12D**	2.88	0.09	5.86	0.95	Undetermined
**1783**	37	F	Primary	**G12V**	3.05	0.81	7.11	0.32	Undetermined
**1461**	80	F	Primary	**G12R**	16.02	0.08	13.37	0.40	**G12R**
**1351**	63	M	Primary	**G12C**	1.58	0.92	2.93	0.87	**WT**
**1781**	55	M	Metastatic	**G13D**	3.69	0.22	6.40	0.13	Undetermined
**1780**	64	M	Primary	**G12V**	2.67	0.40	5.41	0.78	Undetermined
**1241**	54	F	Primary	**G12D**	2.95	0.02	7.18	0.42	**G12D**
**1237**	68	M	Primary	**G13D**	3.03	0.01	5.02	0.30	**G13D**
**1283**	54	M	Primary	**G12D**	3.25	0.06	10.29	0.70	**G12D**
**1584**	62	F	Metastatic	**G12A**	3.85	0.50	9.17	0.67	**WT**
**1350**	69	M	Primary	**G12C**	1.38	0.34	3.52	0.47	**G12C**
**1779**	65	M	Metastatic	No amp by NM ref DNA					Undetermined
**1667**	76	F	Metastatic	No amp by NM ref DNA					Undetermined
**1789**	62	F	Primary	No amp by NM ref DNA					Undetermined
**1675**	68	M	Primary	No amp by NM ref DNA					Undetermined
**1738**	66	F	Primary	No amp by NM ref DNA					Undetermined
**1396**	73	F	Primary	No amp by NM ref DNA					Undetermined
**1782**	59	M	Primary	No amp by NM ref DNA					Undetermined
**1740**	69	M	Primary	No amp by NM ref DNA					Undetermined

### Sensitivity of the KRAS Multiplex q PCR assay

To evaluate the sensitivity and selectivity of the multiplex allele specific assay, reference standard DNAs harboring KRAS mutant was diluted in reference wild-type DNA. The proportion of mutant DNA was gradually reduced to obtain decreasing ratios of mutant to wild-type DNA. We are able to detect in multiplex assay a 0.15% (35 pg) of mutant DNA. The representative example of multiplex allele specific assay using DNA KRAS G12S mutation for limit of detection is shown in S1 Fig. To test the sensitivity of multiplex allele specific assay, KRAS mutations were quantified in DNA mixtures containing mutant DNA and wild type DNA at varying ratios (5%, 2.5%, 1.25%, 0.62%, 0.32%, 0.15%). The results reproducibly detected comparable amount of KRAS mutant alleles in all repeated runs. Mean +/- SD ΔCq values shown in Table 3, indicate the sensitivity of multiplex allele specific assay for each mutation. The ΔCq values of Multiplex assays shown in Table 3 clearly permitted the detection of the more clinically frequent 7 KRAS mutations. However, at low mutant DNA levels, our multiplex assay preferentially identified G12S, G13D and G12C.

**Table 3:** Sensitivity of the KRAS Multiplex qPCR assay.

	G12S		G12V		G12R		G12A		G12C		G12D		G13D	
Input DNA	$\bar{\Delta Cq}$	SD	$\bar{\Delta Cq}$	SD	$\bar{\Delta Cq}$	SD	$\bar{\Delta Cq}$	SD	$\bar{\Delta Cq}$	SD	$\bar{\Delta Cq}$	SD	$\bar{\Delta Cq}$	SD
**5%** (1.12 ng)	3.62	0.27	5.68	0.37	4.74	0.32	4.08	0.72	3.59	0.30	5.89	0.31	2.54	0.14
**2.5%** (0.56 ng)	3.94	0.14	5.4	0.11	4.88	0.63	4.89	0.38	3.55	0.41	6.69	0.13	2.47	0.09
**1.25%** (0.28 ng)	3.99	0.17	5.07	0.17	5.14	0.28	5.13	0.51	4.6	0.73	7.20	0.64	2.84	0.38
**0.62%** (0.14 ng)	3.6	0.60	5.27	0.35	5.75	0.34	5.1	0.11	4.29	0.21	6.71	0.14	3.07	0.76
**0.32%** (70 pg)	3.09	0.22	8.08	0.74	6.23	0.43	4.91	0.2	3.97	0.61	7.72	0.60	3.51	0.64
**0.15%** (35 pg)	**3.54**	0.10	8.62	0.89	5.6	0.12	5.22	0.81	**4.04**	0.39	7.52	0.84	**3.65**	0.49

$\bar{\Delta Cq}$ represents ΔCq mean. Preferential detection of **G12S, G12C, G13D** mutations at lower KRAS levels is indicated by the underlined numbers, with no qPCR amplification evident in the respective NTC (non-template controls).

## Discussion

Since KRAS mutations correlate with resistance to anti-EGFR treatment (cetuximab or panitumumab), there is a need for inexpensive, high throughput assays to detect KRAS mutation in clinical samples [26]. Next generation DNA sequencing (NGS) is highly recommended, but its use is beyond the economic possibilities and expertise of average clinical laboratories. Although Sanger sequencing remains the gold standard for identifying DNA mutations, it is poorly sensitive requiring samples with a tumor cell percentage of 10–30% e.g. due to DNA cross-linked or degraded by formalin fixation, and/or limiting amounts of the target sequence) [27,28].

In Table 2, some FFPE samples failed both in our multiplex assay and by Sanger sequencing. Those tissues that failed to amplify non-mutated DNA controls, came from provincial dispensaries with few resources and limited pathological expertise. Others have shown that DNA extracted from FFPE may be of poor quality, resulting in false-positive or false-negative results due to an incomplete tissue fixation or overfixation [29]. This may cause false negative results that may have unwanted consequences for patient treatment with tyrosine kinase receptors inhibitors [17]. To exclude false negatives, this report used a sensitive detection presence of mutations in codons 12 and 13 of KRAS by allele specific real-time PCR in a multiplex manner concomitantly with detection of a non-mutated KRAS reference control amplicon [22].

Usually, false-positive results are related to cross-reactivity (e.g.,contamination) during tissue isolation or FFPE processing. However, this is diminished by allele-specific PCR, based on the poor initiation of the Taq polymerase from mismatched primers by using primers with 3′ ends that match the KRAS mutations. Since the Taq polymerase does initiate at a low level from mismatched primers, KRAS primers with mutant sequences will sometimes initiate from wild-type sequences, may cause false-positives at high Cq values [30]. Hence, in these studies, allele-specific PCR results are typically considered positive at Cq values below 44 in our uniplex confirmatory assays. Although a similar uniplex assay was reported [22], the latter approach required seven different allele specific PCR assays, demanding consumption of more template DNA and Taqman reagents before mutation detection. In contrast, our multiplex assay simultaneously analyzes 7 mutant primers and probes in the same reaction tube, reducing the amount of clinical material required, which is important in FFPE tissue where the proportion of mutant relative to wild type DNA is likely to be low [22,31,32]. Although another multiplex allele specific PCR employing standard end-point PCR and SYBR green I nucleic acid gel stain was recently used to detect KRAS mutant in clinical samples [10], the latter method requires post-PCR manipulations risking laboratory nucleic acid contamination, since it includes end-point PCR followed by an electrophoretic separation to detect short 64 bp or 68 bp stained products corresponding respectively to the G12S and to the G12V KRAS mutations. Although these small products may be real [10], in some samples they may correspond to non-specific small molecular weight sub-products of end-point PCR, especially since no specific KRAS hybridization probing of this molecule was provided [10]. Moreover, electrophoretic analysis and Sybr Green I stain used [10] limits high throughput analysis. In contrast, our multiplex allele-specific Taqman real-time PCR assays not only has the potential to detect low copy numbers of mutated DNA, but the use of fluorescently labeled probes, increase sensitivity, specificity and reproducibility.

This multiplex allele specific assay potentially allows inexpensive and rapid high throughput screening of the most clinically frequent KRAS mutations in one step, followed a single tube use of seven allele specific primer (i.e, G12S, G12R, G12C, G12D, G12A, G12V and G13D) and common reverse primer and TaqMan probe. In contrast with reports using KRAS plasmid DNA [10,21] to define assay sensitivity, this multiplex allele specific assay was standardized using human reference DNA from clinical samples or from tumor cell lines, permitting multiplex detection of at least 0.15% of mutant DNA (35 pg approximately, equivalent to 5 copies of mutant KRAS DNA) mixed with wild type genomic DNA. Preliminary rapid multiplex mutation detection was followed by precise identification of the specific mutation performed by seven individual allele specific assays. The commercially available Cobas KRAS kit (TaqMelt PCR) test is reported to be <5% sensitivity in KRAS mutations in codon 12/13 and codon 61. On the other hand, the Therascreen KRAS RGQ PCR test reportedly detects mutations at 1% sensitivity in plasmids and cell lines, having limits of detection of 5 to 10 copies and detects seven mutations in codon 12/13 [33].Others indicated that the Therascreen KRAS RGQ PCR kit (Qiagen) or Cobas KRAS kit (TaqMelt PCR) are quite expensive; with the cost per sample may depend on both the type of kit used and on the country where the kit is sold. The cost per sample for the TheraScreen test by 2012 was about USD 143 in USA and has not gone down. In the case of Cobas KRAS test, the cost per sample is about USD 95, additionally requiring equipment other than that used for real-time PCR. The higher cost makes it difficult to use this test in routine clinical practice, with a large number of samples [34]. Other important limitation for use of these kits is their high DNA input requirements (samples with low DNA content), Cobas KRAS test requires 100 ng of total DNA input and Therascreen KRAS RGQ PCR test requires 100–200 ng of total DNA input, with our multiplex assay requiring below 25 ng total DNA [34]. Moreover, in developing countries, the import cost per sample of such tests, are even higher.

### Significance

Unequal KRAS mutations might have different predictive value [28,30]. Although our assay detected KRAS mutations in exon 2, codons 12 and 13 with a ≤ 0.15% sensitivity, this multiplex assay preferentially identified G12S, G13D and G12C at the lowest mutant DNA level. Identification of the G12C mutation is quite important in non-small cell lung carcinoma (NSCL) prognosis, where this mutation is most frequent and correlates with poor prognosis and resistance to anti-tyrosine kinase inhibitor treatment [35] On the other hand, the G13D mutation might have a less detrimental effect on response to cetuximab treatment in CRC [36,37]. In contrast, patients with mCRC tumors harboring G12V or G12A KRAS mutation had the shortest survival observed [31,32,35]. Taken together, our assay might be used for identification of KRAS status in either CRC or NSCLC [37–39].

## Supporting Information

S1 Fig Sensitivity of Multi-AsP qPCR assay. Panel A show the amplification curves of Mult-AsP assay, as the % of mutant G12S DNA to wild-type DNA at 5%, 2.5%, 1.25%, 0.62%, 0.32% and 0.15%. Wild-type KRAS DNA (HD135, Horizon) was used as non-mutated control. Panel B shows the Cq values of each reaction. This suggests that the Mult-AsP has the sensitivity to detect 35 pg, relative to gray curves indicated NTC reaction.
(TIF)

S1 Table Primers and Probe. The probe for *KRAS* was labeled with FAM at the 5′ end and an MGB at the 3′ end. Artificial mismatches are indicated as lowercases [22].
(DOCX)
