# Supplementary figures and images for: Optimized Multiplex Detection of 7 KRAS Mutations by Taqman Allele-Specific qPCR
Source: PLoS One. 2016 Sep 15;11(9):e0163070. doi: 10.1371/journal.pone.0163070 (PMC5025196; doi:10.1371/journal.pone.0163070)

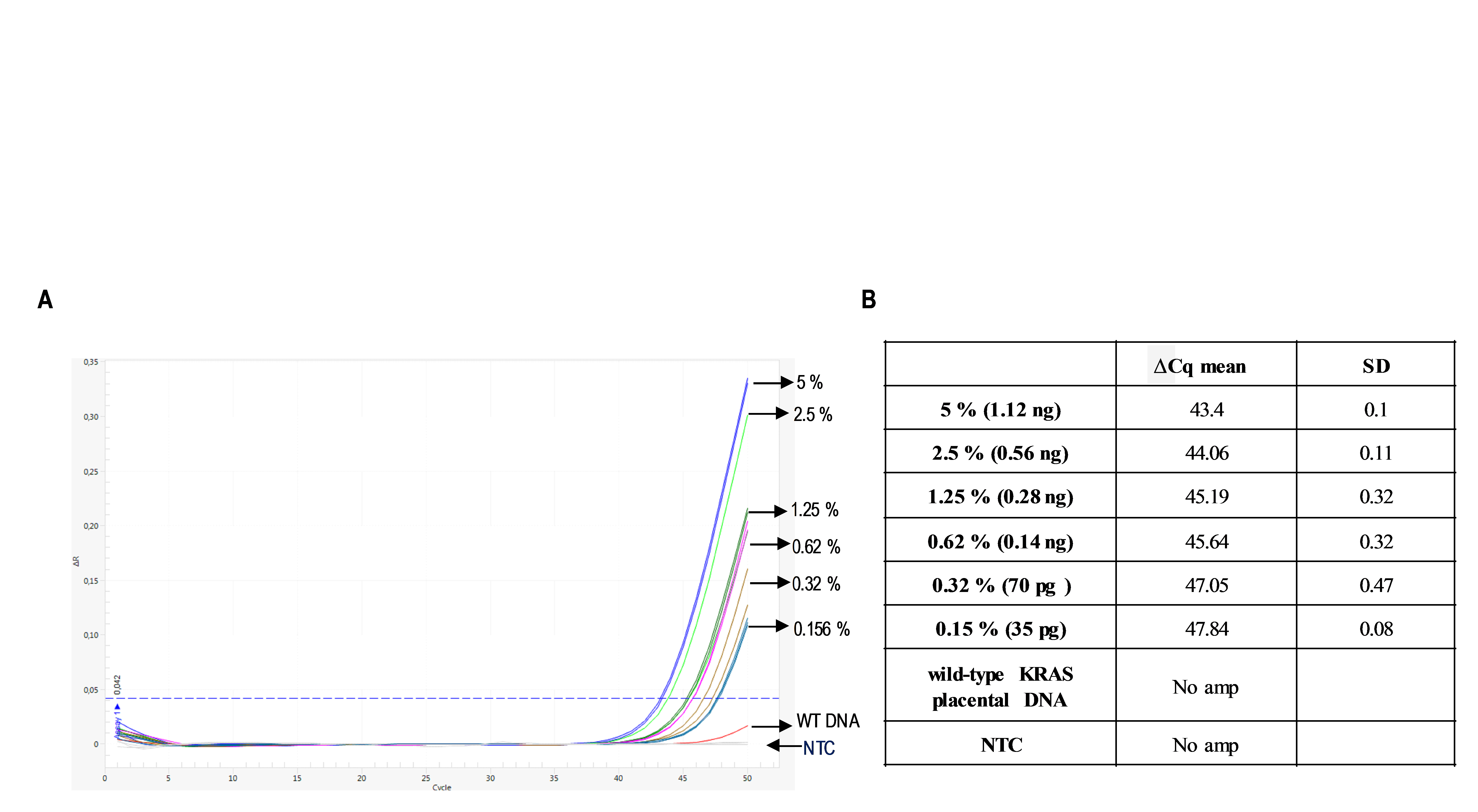

Supplement: S1 Fig — Panel A show the amplification curves of Mult-AsP assay, as the % of mutant G12S DNA to wild-type DNA at 5%, 2.5%, 1.25%, 0.62%, 0.32% and 0.15%. Wild-type KRAS DNA (HD135, Horizon) was used as non-mutated control. Panel B shows the Cq values of each reaction. This suggests that the Mult-AsP has the sensitivity to detect 35 pg, relative to gray curves indicated NTC reaction. (TIF) [file pone.0163070.s001.tif]
