# Supplementary material for: Optimized Multiplex Detection of 7 KRAS Mutations by Taqman Allele-Specific qPCR
Source: PLoS One. 2016 Sep 15;11(9):e0163070. doi: 10.1371/journal.pone.0163070 (PMC5025196; doi:10.1371/journal.pone.0163070)
Supplement: S1 Table — The probe for KRAS was labeled with FAM at the 5′ end and an MGB at the 3′ end. Artificial mismatches are indicated as lowercases [22]. (DOCX) [file pone.0163070.s002.docx]

**S1Table. Primers and Probe.**

| **Primer** | **Sequence** |
| --- | --- |
| G12A | 5´- AACTTGTGGTAGTTGGAGCTtC-3´ |
| G12S | 5`-AATATAAACTTGTGGTAGTTGGAGCgA-3´ |
| G12C | 5´- AATATAAACTTGTGGTAGTTGGAGCcT-3´ |
| G12V | 5´- AAACTTGTGGTAGTTGGAGCaGT-3´ |
| G12R | 5´-AATATAAACTTGTGGTAGTTGGAGCTC-3´ |
| G12D | 5´- AAACTTGTGGTAGTTGGAGCgGA-3´ |
| G13D | 5´- GTGGTAGTTGGAGCTGGaGA-3´ |
| Reference Fw | 5´- GACTGAATATAAACTTGTGGTAGTTGGA-3´ |
| Rv KRAS | 5´- CATATTCGTCCACAAAATGATTCTG-3´ |
| Probe | 5´- FAM-CTGTATCGTCAAGGCACT-MGB-3´ |

The probe for KRAS was labeled with FAM at the 5′ end and an MGB at the 3′ end. Artificial mismatches are indicated as lowercases [22].
